# Ketamine Dependence: Clinical Characteristics of an Inpatient Detoxification Population With Complex Physical Health Needs

**DOI:** 10.1192/bjo.2026.11591

**Published:** 2026-06-30

**Authors:** Sarah Shackleton, Onuswale Oni, Stephen Kaar

**Affiliations:** 1 University of Manchester, Manchester, United Kingdom; 2 Lancashire and South Cumbria NHS Foundation Trust, Preston, United Kingdom; 3 Greater Manchester Mental Health NHS Foundation Trust, Manchester, United Kingdom

## Abstract

**Aims:**

Recreational ketamine use and treatment-seeking for associated complications is increasing in England. Complications of ketamine dependence can be debilitating, leading to continued dependence and surgical intervention. However, treatment remains under-researched, with lacking understanding of those seeking treatment or requiring specialist inpatient detoxification. This study aims to analyse demographic and clinical characteristics of an inpatient ketamine detoxification population in the north of England between January 2016 and September 2025, with the aim to inform public health strategies and aid prevention efforts.

**Methods:**

This retrospective cross-sectional study was conducted using routinely collected clinical data for patients that attended for ketamine detoxification at the Chapman Barker Unit (CBU), a regional specialist inpatient detoxification treatment service in Greater Manchester, UK. Patients were identified using clinical logbooks and the Patient Registration and Information System (PaRIS). The primary analysis included 59 patients admitted for ketamine detoxification. Comparative analyses were conducted between the ketamine-only detoxification patients (n=23) and the ketamine plus polysubstance detoxification patients (n=36) to explore the relationship between ketamine dose and physical harms.

**Results:**

Ketamine detoxification admissions increased between 2016 and 2025. One third occurred between January and August 2025. Most patients were male, younger, WhiteBritish, heterosexual, single and unemployed with traumatic experiences and criminal justice involvement. Mean age of ketamine use was 18 years with 5.2 years of usage. Most inhaled ketamine, and mean daily usage was 5.4g, with higher dose in those experiencing lower urinary tract symptoms. Anxiety, depression, suicidal ideation and past suicide attempts were common, along with concerning urological pathologies in particular ketamine bladder syndrome, cystitis, nasal damage and hydronephrosis. Cravings and anxiety were common withdrawal symptoms. Alanine transaminase (ALT) levels increased from admission to discharge. Ketamine-only detoxification patients were more likely to be single, use higher ketamine doses, inhale ketamine, experience depression, have ketamine bladder syndrome and hydronephrosis. PTSD was more prevalent in polysubstance patients. Diazepam reducing regimes were the standard detoxification approach. Most patients completed detoxification.

**Conclusion:**

Ketamine detoxification admissions are increasing, with a predominantly young population with prolonged high-dose ketamine usage, associated urological pathology and hepatic injury. Specialist inpatient detoxification is successful, and further evidence-based treatment and relapse prevention protocols are required. Targeted public health campaigns should raise awareness of the physical, psychological and social harms associated with ketamine dependence.

